# Prolonged survival in HIV-associated Progressive Multifocal Leukoencephalopathy treated with Pembrolizumab: a case series on treatment and long-term follow-up

**DOI:** 10.1007/s13365-025-01243-0

**Published:** 2025-02-19

**Authors:** Marta Chiuchiarelli, Giulia Micheli, Francesco Vladimiro Segala, Gabriele Giuliano, Paola Del Giacomo, Alex Dusina, Elena Matteini, Federico Frondizi, Simona Gaudino, Francesca Lisi, Eleonora Cimini, Rosaria Santangelo, Chiara Agrati, Carlo Torti, Antonella Cingolani

**Affiliations:** 1 Dipartimento di Sicurezza e Bioetica - Sezione di Malattie Infettive, Università Cattolica del Sacro Cuore, Rome, Italy; 2 Clinical Infectious Diseases Department, National Institute for Infectious Diseases Lazzaro Spallanzani IRCCS, Rome, Italy; 3 Clinic of Infectious Diseases, Department of Precision and Regenerative Medicine and Ionian Area (DiMePRe-J), University of Bari Aldo Moro, Bari, 70124 Italy; 4 Department of Medical Biotechnologies, University of Siena, Siena, Italy; 5 Infectious and Tropical Diseases Unit, Azienda Ospedaliero-Universitaria Senese, Siena, Italy; 6 Dipartimento di Scienze Mediche e Chirurgiche, Fondazione Policlinico Universitario Agostino Gemelli IRCCS, Rome, Italy; 7 Clinic of Infectious Diseases and Tropical Medicine, San Paolo Hospital, ASST Santi Paolo e Carlo, Department of Health Sciences, University of Milan, Milan, Italy; 8 Diagnostic Neuroradiology Unit, Fondazione Policlinico Universitario Agostino Gemelli, IRCCS, Largo A. Gemelli, 8, Rome, 00168 Italy; 9 Laboratory of Cellular Immunology and Pharmacology, National Institute for Infectious Diseases, “L. Spallanzani”, Rome, Italy; 10 Institute of Microbiology, Catholic University School of Medicine, Largo F. Vito, 1, Rome, 00168 Italy; 11 Unit of Pathogen Specific Immunity Department of Pediatric Hematology and Oncology, IRCCS Ospedale Pediatrico Bambino Gesù, Rome, Italy

**Keywords:** PML, HIV, Pembrolizumab, PD-1 inhibitors

## Abstract

Progressive Multifocal Leukoencephalopathy (PML) is a rare opportunistic infection of the central nervous system (CNS) caused by human polyomavirus JC virus, with high mortality rate in people living with HIV (PLWH), without an effective specific treatment beside combined antiretroviral therapy (cART). The use of Pembrolizumab, an inhibitor of the Programmed cell death protein 1 (PD-1) receptor on T cells, has been associated with decreased viral clearance. Aim of this study is to evaluate clinical course of PLWH affected by PML treated with pembrolizumab. We report four consecutive PLWH with clinical and radiological evidence of PML and JCV-DNA detection in cerebrospinal fluid (CSF). Pembrolizumab was administered to all four PLWH alongside cART. Radiological and laboratory follow-up were performed at the end of the medical protocol. Clinically, 3 out of 4 PLWH showed an improvement in neurological deficits, partially reacquiring the lost functions, and they are alive at 3.5 years, 14 months, and 9 months, respectively; the fourth patient died shortly after treatment due to worsening respiratory conditions. In all the PLWH completing treatment, a decrease of about 80–90% of the specific PD-1 activity was observed. Prolonged survival and stabilization of radiological findings have been observed, along with clinical improvement and partial recovery of acquired deficits in 3 out of 4 PLWH. In addition, a decrease in anti-PD-1 expression has also been observed, suggesting a link between the therapy and the success achieved. Given the small sample and conflicting evidence in the existing literature, further investigation is needed to assess its effectiveness.

## Introduction

Progressive Multifocal Leukoencephalopathy (PML) is a rare and severe neurological complication in people with HIV (PLWH) and many other immunosuppressed patients, caused by JC virus.

PML’s landscape has undergone significant transformations recently, driven by novel therapeutic approaches in many domains, changing the current epidemiology to further at-risk populations (Möhn et al. 2021). As the population at-risk of PML expands with medical progress, so it does the need for innovative treatment strategies (Cortese et al. 2019). There is no currently approved therapy yet, but, in recent years, immunotherapy has emerged as a promising frontier in the management of PML, offering new hope and challenges to clinicians and researchers (Baena-Álvarez et al. 2022).

Pembrolizumab (PEM) is a monoclonal antibody that inhibits the programmed cell death protein-1 (PD-1), which is currently approved for salvage therapy in many oncological conditions. PD-1 expression, an adverse immune regulator expressed on activated T cells, is upregulated on CD4 + and CD8 + cells of patients with PML, particularly on JC virus-specific CD8 + T cells. It has been theorized recently that the inhibition of PD-1 could be associated with better anti-JCV-specific response, with consequent JCV clearance and neurological improvement (Pinnetti et al. 2022; Boesl et al. 2022; Holmes et al. 2020). Two ongoing trials (e.g., NCT04091932, NCT06276504), have been registered in 2019 and 2024 with the aim to evaluate its safety and efficacy, particularly in reducing JC virus replication. However, no conclusive data are currently available, and the use of pembrolizumab for PML remains experimental. Further research is needed to establish its therapeutic role (National Library of Medicine n.d.; Assistance Publique - Hôpitaux de Paris n.d.).

In this intricate scientific landscape, despite the utilization in recent years of several antiviral and non-antiviral therapeutic strategies, PML remains a condition that still needs a viable therapeutic strategy. According to the latest European and international guidelines, the sole “therapy” for PML is the elimination of the underlying immunosuppressive state, which, within the context of HIV infection, translates to an effective combination antiretroviral therapy (cART) (Lambert et al. 2021; Redelman-Sidi et al. 2018; Wicklein et al. 2021).

In light of these statements and the literature reports, our objective is to describe the experience gained through implementing the use of pembrolizumab as a rescue therapy in cases of PML treated at our University hospital. We hereby describe clinical outcomes in terms of survival and improvement of neurological deficits, JCV-DNA quantitative reduction, PD-1 downregulation, and enhanced JCV-specific T-cell response after pembrolizumab treatment in PLWH affected by PML (Cortese et al. 2019; Pinnetti et al. 2022).

## Materials and methods

We describe our experience on four consecutive PLWH diagnosed with PML (three males and one female) observed between 2019 and 2023, treated with Pembrolizumab as off-label prescription. PLWH received a treatment protocol consisting of at least four intravenous infusions of Pembrolizumab at a dose of 2 mg/kg, given every month along with daily cART. All PLWH had detectable JC virus (JCV)-DNA in cerebrospinal fluid (CSF) at baseline.

During their care, an MRI imaging was performed at baseline in all PLWH to assess the extent and characteristics of lesions associated with PML, along with laboratory testing including baseline biochemical profile, quantitative serum HIV-RNA and PCR for JC virus in serum. Moreover, several assays were performed on CSF obtained through lumbar puncture (LP): aerobic and anaerobic cultures for bacteria and fungi, quantitative HIV-1-RNA, and PCR for JC-virus. In addition, PD-1 expression on circulating and in CSF CD4 and CD8 T lymphocytes was performed on whole blood and CSF samples by flow cytometry. Briefly, 100 ul of whole blood was stained with a cocktail of mAbs (CD3 PE, CD8-FITC, CD4 APC, PD-1 Percp-Cy5. (BD Biosciences) for 20 min at + 4 °C. Whole blood was then washed with buffer (PBS 1×, 0.1% NaN3, 1% BSA), fixed with 100 ul of Paraformaldehyde (PFA, Sigma) and acquired to a cytometer (DxFlex, Beckman Coulter). Peripheral blood mononuclear cells (PBMC) from PLWH were isolated by gradient centrifugation (Lympholyte, Cedarlane, Canada), counted with Trypan blue and frozen in FBS (Fetal Bovine Serum, Euroclone, Italy) added of 10% DMSO (Euroclone, Italy). Subsequently, PBMC of PLWH were thawed and suspended 1 × 10^6^ cells/mL of complete medium [RPMI-1640 supplemented with 10% FBS, 2 mmol glutamine, 50 IU/mL penicillin, and 50 µg/mL streptomycin (Corning)], counted by Trypan blue exclusion and used for the experimental design. To analyze JCV T cells specific response, we stimulated PBMC from PLWH with JCV specific peptides (specific for VP1 and LT1 JCV proteins, 2ug/ml) for 20 h at 37 °C in 5% of CO2. Anti-CD28 and anti-CD49d (1ug/ml, BD Biosciences, USA) were added in culture. As a positive control (the evaluation of the immunocompetence), PBMC were stimulated with phytohemagglutinin (PHA). At the end of incubation, the ELISpot assay was developed according to the kit’s instructions (Mabtech, Sweden). Spontaneous cytokine production (background) was assessed by incubating PBMC with complete medium. Results are expressed as spot-forming cells (SFC) per 10⁶ PBMCs in stimulating cultures after subtracting the background. These investigations were repeated during the treatment (about one month from the first infusion) and at the end, whenever possible. Data collected from baseline assessments, including imaging results and laboratory findings, were analyzed. Descriptive statistics were employed to summarize demographic characteristics, and changes in clinical parameters were assessed over the treatment period, whose data are described in Table 1. All participants provided informed consent.

**Table 1:** PLWH demographic and clinical characteristics

PT	Sex	Age	CD4/mmc	HIV-RNA at PML onset (cp/ml)	Comorbidities	Nr of Pembrolizumab doses	Outcome
1	M	71	372	0	NH B-cell lymphoma	8	Alive at 3.5 y/clinical and MRI improvement
2	F	41	8	8279	HCV infection	4	Alive at 14 mts/ clinical and MRI improvement
3	M	43	91	0	Neurotoxoplasmosis	4	Alive at 9 mts/clinical and MRI stable
4	M	51	103	327,208	Disseminated CMV	2	deceased due to worsening of neurological conditions and pneumonia

## Results

Patient 1, a 71-year-old HIV-positive male with a baseline CD4 count of 372/mmc and undetectable plasma HIV-RNA under successful cART with tenofovir alafenamide/emtricitabine/darunavir/cobicistat + dolutegravir (TAF/FTC/DRV/c + DTG) due to a previous virological rebound without resistance on TAF/FTC + D. He was recovering from non-Hodgkin B-cell lymphoma when the diagnosis of PML was formulated. The MRI showed a T2 and FLAIR hyperintensity in the white matter of the left frontal lobe (superior and middle frontal gyri, and precentral gyrus), predominantly involving the arcuate fibers and extending to the centrum semiovale and corona radiata, along with a linear, gyriform hypointense rim on Susceptibility Weighted Imaging (SWI), involving the subcortical frontal region. There was no mass effect, and only a faint peripheral patchy contrast enhancement. These MRI findings were typical of PML. The LP was not examined for PD-1 expression in T cells at baseline. PD-1 expression in the CSF and whole blood (WB) remained undetectable throughout the study period when examined. The patient showed no cells expressing PD-1 in CSF at 4–8 months from baseline interval, while in WB, there was an increase from CD8 + 0.1%, CD4 + 0.5% at baseline to CD8 + 1.6%, CD4 + 2.3% at 4–8 months. This patient received eight administrations of Pembrolizumab on a monthly basis, with a scheme partially different from the other PLWH in the case series, due to the lack of evidence of a precise length of the experimented regimen at the time, the absence of toxicities and the good clinical response. Along with the pembrolizumab injection, he also underwent intensive speech therapy and neuropsychological evaluations several times during the follow-up period. A two-year follow-up assessment by neuropsychologists showed improvement in complex mnesic mechanisms and speech production evaluation. Notably, the patient was alive at 3.5 years with clinical and MRI three years from baseline showed stability of all brain lesions, lack of contrast enhancement, and in the absence of signs of disease reactivation, fully autonomous in activities of daily living.

Patient 2, a 41-year-old female, was diagnosed with HIV infection at the same time of the PML presentation. She had a baseline CD4 count of 8/mmc and baseline serum HIV-RNA of 8279 copies/ml, along with HCV co-infection. At baseline, she presented with progressive speech impairment, balance deficits and ataxia. Baseline MRI showed the presence of bilateral and symmetrical hyperintensities on FLAIR and T2 with corresponding hypointensities on T1, involving the bilateral cerebellar hemispheres, vermis, and bilateral middle cerebellar peduncles, without contrast enhancement and mass effect. Also, JCV-DNA was detected at CSF examination. Taken into account the history, imaging findings could be compatible with JCV granule cell neuronopathy (JCVGCN). Also, JCV-DNA was detected at CSF examination. She was promptly started on cART and pembrolizumab. During treatment period, there were significant changes in JCV-specific PD-1 expression, with decreased activity in CSF and WB from baseline to 4–8 months: despite initial high expression levels, CD8 + and CD4 + PD-1 expression decreased from 85.5% and 86.7–72.3% and 43.9% in WB at 4–8 months, respectively. Regarding JCV specific T cells response, we observed a non-detectable response at baseline, while an improvement was observed after the first dose of pembrolizumab administration (127 IFNg SFC/10^6^ PBMC) until 4–8 months (100 IFNg SFC/10^6^ PBMC). The patient was alive at 19 months with clinical improvement, recovering partially from production aphasia and reacquiring partial motor abilities.

**Fig. 1:**
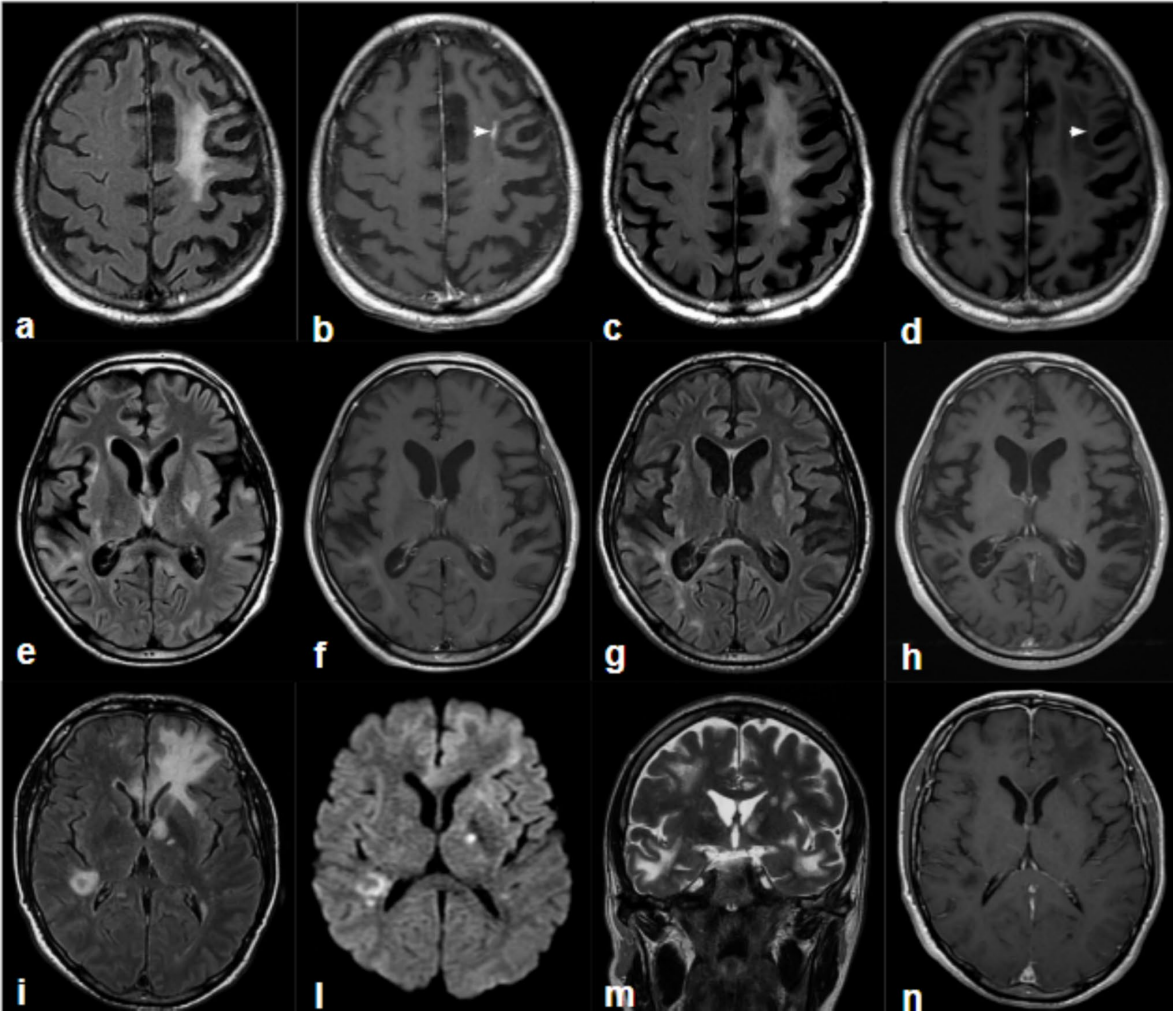
MRI imaging. **Patient 1 (first Row, a-d)**: FLAIR (**a**) and T1-weighted post-contrast images (**b**) from the first MRI exam, and the corresponding FLAIR (**c**) and T1-weighted post-contrast images (**d**) after three years. The initial exam shows a FLAIR hyperintensity in the white matter of the left frontal lobe, involving the subcortical U fibers, with no mass effect and faint marginal enhancement (arrow in b), consistent with typical PML findings. Over time, the FLAIR lesion remains stable, while contrast enhancement resolves (arrow in d). **Patient 3 (second Row, e-h)**: FLAIR (**e**) and T1-weighted post-contrast images from the first MRI exam, and the corresponding FLAIR (**g**) and T1-weighted post-contrast images (**h**) after 12 months. The initial FLAIR images reveal bilateral, patchy, ill-defined cortical and subcortical hyperintense lesions without mass effect; some lesions exhibit faint marginal enhancement. These imaging findings are atypical for PML. The follow-up MRI shows a reduction in lesion size, persistent FLAIR hyperintensities, and evidence of ventricular and sulcal expansion. **Patient 4 (third row, i-n)**: Single MRI exam with FLAIR (**i**), DWI (**l**), coronal T2 (**m**), and T1-weighted post-contrast (**n**) images. The MRI demonstrates multiple FLAIR hyperintense lesions involving the white matter of multiple bilateral lobes, the splenium, left internal capsule, and nucleus pallidus. DWI reveals variable signal intensities: lesions in the left frontal and right periventricular regions show a low-signal core surrounded by a high-signal rim, with a punctate high signal in the globus pallidus and intermediate-to-low signal in the corpus callosum. Coronal T2 imaging shows a slight mass effect on the left ventricle, which appears reduced in size compared to the contralateral side. No contrast enhancement is observed

Patient 3, a 43-year-old male diagnosed with HIV infection in 2022, with a baseline CD4 count of 91/mmc and undetectable plasma HIV-RNA under 4 months cART with BIC/TAF/FTC, was recovering from a recent episode of neurotoxoplasmosis, for which he underwent effective treatment with sulfadiazine and pyrimethamine. After recovering from the disease, he experienced an episode of neurological worsening, with the new onset of tremors, confusion, and production aphasia so he was transferred in our facility and an MRI was performed, showing bilateral, patchy cortical and subcortical hyperintense lesions. There was no mass effect. T1 images after contrast administration showed marginal enhancement of some lesions. These MR imaging findings were not typical for PML. At baseline, CD4 + and CD8 + PD-1 expression in CSF and WB were very high (86% and 87% in CSF and 55.5% and 77.7% in WB, respectively) showing variations during the study period, with an initial high expression followed by a decrease at 1–4 months in circulating CD4 and CD8 T cells (50.2% and 36.8%, respectively). Unfortunately, this decrease did not correspond to an improvement of T cells specific response. Notably, lumbar puncture was not performed for 4–8 months. The patient was alive at 12 months follow-up. Final MRI showed reduction in lesion size, persistent FLAIR hyperintensities, and evidence of ventricular and sulcal expansion as for a mild cerebral atrophy. He also experienced a clear clinical improvement, reacquiring independence in activities of daily living. Patient 4, a 51-year-old male, was diagnosed with PML at the same time as HIV diagnosis, with a baseline CD4 count of 103/mmc and a plasma HIV-RNA of 327,208 copies/mL; also JCV virus in blood was of 1169000 cp/ml. During hospitalization, he also presented with systemic CMV infection, with a CMV-DNA PCR of 332556 cp/ml, fever, treated with intravenous ganciclovir, cART and pembrolizumab. PD-1 high expression in circulating and in CSF CD4 + and CD8 + T cells was showed at 1–4 months. After 1 dose of pembrolizumab, the patient died 60 days after PML diagnosis, due to worsening neurological status and respiratory complications. An MRI performed ten days before death showed increase -compared to the previous MRI- extension of the multifocal hyperintensity in T2 and FLAIR images involving the subcortical and deep white matter of the frontal and temporal lobes bilaterally, the left insular cortex, the cingulate gyrus on both sides, the left nucleus-capsular region, the corpus callosum, both cerebral peduncles, the pons, and the left middle cerebellar peduncle. On DWI larger lesions showed slightly restricted diffusion along its margins, and some little lesions showed high restriction, the appearance on DWI probable varied with lesions stage. No contrast enhancement was observed. Of interest, some lesions had a slight mass effect. Table 2 summarizes immunological values on circulating and in CSF CD4 and CD8 T cells of enrolled PLWH. Meanwhile, Fig. 1 reports PLWH’ MRI pattern throughout the study.

**Table 2:** Immunological parameters

Patients	Pt1	Pt2	Pt3	Pt4
**Baseline**				
CD4 + PD1 + WB (%)	ND	86.7	77.7	98.8
CD8 + PD1 + WB (%)	ND	85.5	55.5	95.9
CD4 + PD1 + CSF (%)	ND	85.6	87.5	90
CD8 + PD1 + CSF (%)	ND	89.2	86.8	82.4
IFNγ SFC/10^6^ PBMC	ND	0	0	0
**1–4 months**				
CD4 + PD1 + WB (%)	0.5%	0	50.2	Deceased
CD8 + PD1 + WB (%)	0.1%	0	36.8	
CD4 + PD1 + CSF (%)	ND	49.2	87.5	
CD8 + PD1 + CSF (%)	ND	72.3	79.3	
IFNγ SFC/10^6^ PBMC	ND	127	0	
**4–8 months**				
CD4 + PD1 + WB (%)	2.3%	49.6	No samples available	
CD8 + PD1 + WB (%)	1.6%	60		
CD4 + PD1 + CSF (%)	ND	ND		
CD8 + PD1 + CSF (%)	ND	ND		
IFNγ SFC/10^6^ PBMC	ND	100		

*ND *Not done for absence of cells in CSF SFC: Spot Forming Cells

The percentage (%) of PD-1 + CD4 and CD8 T cells in the whole blood (WB) and in CSF was evaluated by flow cytometry. The JCV-specific T cell response was evaluated by Elispot assay and expressed as spot forming cells (SFC) / 106 PBMC

## Discussion

PML in PLWH carries a high burden of mortality and morbidity and it still has no defined effective therapeutic strategies. Immunotherapy, specifically pembrolizumab, an immune checkpoint inhibitor targeting programmed cell death protein 1 (PD-1), has shown promise in PML management (Cortese et al. 2019). First evidence supporting the use of pembrolizumab for treating PML, showcasing its potential as a therapeutic agent, appeared in 2019 (Möhn et la. 2021; Cortese et al. 2019). Recently, this monoclonal antibody has also been tested in different study groups and protocols, including not only PLWH, but also diverse severely immunocompromised populations, displaying its potential in controlling PML progression by modulating the immune response against the JC virus (Cortese et al. 2019; Pinnetti et al. 2022; Boesl et al. 2022; Holmes et al. 2020). An Italian case series delved into its application in PLWH: checkpoint inhibitors-therapy was successful in 3 out of 5 PLWH, with no progression of the clinical findings (Pinnetti et al. 2022). Moreover, a Chinese case series with a similar sample also provided evidence of positive outcomes in PLWH treated with pembrolizumab (2–4 doses administered) (Ambrosioni et la. 2023). In addition, in 2023 a report from Kings’ College in London described a rare case of idiopathic CD4 T-cell lymphocytopenia presenting with PML, treated successfully with 3 doses of pembrolizumab, with the complete remission of disease (Panel on Antiretroviral Guidelines for Adults and Adolescents n.d.). Recent case reports suggested novel approaches to PML treatment using sequential treatment strategy involving intravenous immunoglobulins and pembrolizumab or combining pembrolizumab and BK virus-specific T cells (Boesl et al. 2022; Lambert et al. 2021). Our study builds on this emerging evidence, giving insights into the dynamics of JCV-specific PD-1 expression, treatment response, and clinical outcomes in PLWH with PML treated with pembrolizumab. The first case, marked by a prolonged history of successful antiretroviral therapy and concurrent NH B-cell lymphoma, is quite unique and highlights the central role of a severely compromised immune system as a risk factor of PML, not due solely to the presence of severe HIV but of the combination of immunological diseases of other origin to the CD4 T-cell depletion of advanced HIV infection. Sustained clinical and MRI improvement over a 3.5-year period was demonstrated for this patient after pembrolizumab use. Despite a different kind of laboratory finding for the patient, due to the fact there was less evidence on the disease and on the treatment protocol and some test were not performed at the time, the notable increase in CD8 + and CD4 + T cells expressing PD-1 in WB further points out the important role of systemic immune response in overcoming JCV CNS infection. This suggests that Pembrolizumab may enhance anti-JCV-specific responses, contributing to JCV clearance (Cortese et al. 2019; Pinnetti et al. 2022). Furthermore, although this consideration is beyond the scope of the present study, the evolution of this patient could suggest that physical therapy in terms of speech therapy and rehabilitation/occupational therapy is crucial in the follow-up of PLWH with PML. Patient 2, presenting with a low baseline CD4 count and HCV co-infection, exhibited a decrease in JCV-specific PD-1 expression in both CSF and WB over the 4–8-month period. This reduction in PD-1 expression coincided with clinical improvement, and recovery from production aphasia and partial motor ability showing. Anyway, even in this patient, pembrolizumab may have modulated the immune response and mitigated PML progression (Pinnetti et al. 2022; Boesl et al. 2022). In Patient 3, the fluctuating JCV-specific PD-1 expression, clinical improvement, and stable MRI findings suggest a dynamic interplay between immunomodulation and disease resolution. The absence of lumbar puncture data at 4–8 months poses challenges in fully interpreting the findings. Nonetheless, the patient’s overall improvement, regaining independence in activities of daily living, emphasizes the potential benefits of Pembrolizumab in neuroinvasive diseases as already reported in literature (Pinnetti et al. 2022; Boesl et al. 2022). Patient 4, unfortunately, died due to worsening neurological status and respiratory complications, in the context of disseminated CMV, PML and newly diagnosed HIV infection. The dynamic pattern of PD-1 expression, with an initial increase followed by a subsequent decrease, suggests a complex immunological response, in particular, it cannot be ruled out that concomitant CMV infection may have altered immunological homeostasis and any potential response to pembrolizumab therapy.

It has been observed that patients with PML display higher percentages of PD-1 + CD4 + and CD8 + T cells in the CSF and blood (Cortese et al. 2019) and increased expression of PD-1 on JCV-specific CD8 + cytotoxic T-lymphocytes (Yang et al. 2023). Moreover, it has been demonstrated that blocking PD-1in vitro resulted in an increase in IFN-γ expression in JCV-stimulated CD8 + T cells in the setting of HIV- associated PML (Varmpompiti et al. 2023). On the other hands, the interplay between HIV and JCV is complicated by the risk of developing immune reconstitution inflammatory syndrome (IRIS), which represent a dysregulated, hyper-inflammatory response potentially resulting in immune-mediated damage, and in a worsening of PML-related neurological outcomes (Hoang et al. 2019). Even though cases of IRIS- PML could be ruled out from our case series, it should be pointed out that an optimal response to checkpoint inhibitors in HIV-associated PML can result from a balance between JCV burden and appropriate degree of immune response.

This case series is in line with the moderately encouraging results from previous studies even though it has some limitations, being a single-center study conducted on a very small sample of PLWH and using retrospective data: it was not possible to obtain all the samples from some PLWH or no serial lumbar punctures were performed for clinical and technical reasons; in patient 1 for instance we do not possess PD-1 expressions in CSF T cells at baseline, and patient 2 lacks of follow-up MRIs. However, the accumulating data on the use of pembrolizumab in PLWH, including the present study, suggest that the drug may have a positive effect on clinical outcome in selected PLWH with high specific PD1 expression. The assessment of such activity in the context of newly diagnosed HIV infection rather than concomitant opportunistic infections may be complex and burdened by confounding factors that make its interpretation and response to therapy problematic. Further studies are warranted to delineate the factors contributing to differential treatment responses observed in PLWH with several infectious comorbidities (Pinnetti et al. 2022; Boesl et al. 2022; Holmes et al. 2020).

## Conclusion

The current therapeutic landscape for PML primarily revolves around eliminating underlying immunosuppression, especially in the context of HIV infection (Tan et al. 2012; Kartau et al. 2019). With its modulatory action on immune responses, Pembrolizumab presents a promising addition to the armamentarium against PML. Despite its limitations, our study provides valuable insights into the individualized responses to Pembrolizumab, emphasizing the need for tailored treatment approaches. The reported synergistic effects of combining Pembrolizumab with other immunomodulatory agents in the literature also warrant exploration in future studies. Clinical trials are needed to investigate specific treatments for PML in HIV in larger cohorts.

## Data Availability

No datasets were generated or analysed during the current study.
